# Stress memory induced rearrangements of *HSP* transcription, photosystem II photochemistry and metabolism of tall fescue (*Festuca arundinacea* Schreb.) in response to high-temperature stress

**DOI:** 10.3389/fpls.2015.00403

**Published:** 2015-06-16

**Authors:** Tao Hu, Shu-Qian Liu, Erick Amombo, Jin-Min Fu

**Affiliations:** Key Laboratory of Plant Germplasm Enhancement and Specialty Agriculture, Wuhan Botanical Garden, Chinese Academy of SciencesWuhan, China

**Keywords:** metabolite profiles, tall fescue, high-temperature stress, chlorophyll a fluorescence (OJIP), HSP, transcriptional memory, heat acclimation

## Abstract

When plants are pre-exposed to stress, they can produce some stable signals and physiological reactions that may be carried forward as “stress memory”. However, there is insufficient information about plants' stress memory responses mechanisms. Here, two tall fescue genotypes, heat-tolerant PI 574522 and heat-sensitive PI 512315, were subjected to recurring high-temperature pre-acclimation treatment. Two heat shock protein (HSP) genes, *LMW-HSP* and *HMW-HSP*, exhibited transcriptional memory for their higher transcript abundance during one or more subsequent stresses (S2, S3, S4) relative to the first stress (S1), and basal transcript levels during the recovery states (R1, R2, and R3). Activated transcriptional memory from two trainable genes could persist up to 4 days, and induce higher thermotolerance in tall fescue. This was confirmed by greater turf quality and lower electrolyte leakage. Pre-acclimation treatment inhibited the decline at steps of O-J-I-P and energy transport fluxes in active Photosystem II reaction center (PSII RC) for both tall fescue genotypes. The heat stress memory was associated with major shifts in leaf metabolite profiles. Furthermore, there was an exclusive increase in leaf organic acids (citric acid, malic acid, tris phosphoric acid, threonic acid), sugars (sucrose, glucose, idose, allose, talose, glucoheptose, tagatose, psicose), amino acids (serine, proline, pyroglutamic acid, glycine, alanine), and one fatty acid (butanoic acid) in pre-acclimated plants. These observations involved in transcriptional memory, PSII RC energy transport and metabolite profiles could provide new insights into the plant high–temperature response process.

## Introduction

When plants are exposed to environmental stresses, they respond complexly, which may involve extreme changes in physiological and gene expression patterns (Molinier et al., 2006). A recurring pre-exposure to biotic or abiotic stress may produce appropriate alarm signal and faster reactions memorized by plants and recognized in subsequent stress (Chinnusamy and Zhu, 2009; Ding et al., 2012). Although most of these stress-induced signal and reactions on physiology and gene expression are reset to the basal level once the stress is relieved, they may be stable and carried forward as a form of “stress memory” (Goh et al., 2003; Chinnusamy and Zhu, 2009; Liu et al., 2014). The physiological traits and gene expression profile changes may contribute to the plants' stress memory. Previous studies have shown that stress memory induced by environmental stresses pre-exposure such as drought stress, cold, and hormones may enhance plants immunity system to enable them cope more effectively with subsequent stress (Goh et al., 2003; Thomashow, 2010; Ding et al., 2012; Liu et al., 2014). Ding et al. (2012) primarily reported that a repetitive dehydration/rehydration pretreatment could induce the transcriptional memory of trainable genes which helped to slow down the wilting of *Arabidopsis* leaves under the subsequent drought stress. However, the mechanisms of stress memory responses involved in physiological and molecular regulation are highly complex and insufficiently documented. The photosynthetic process changes or accumulation of metabolic profiles may be proposed to understand the stress memory responses.

Photosystem II (PSII) as the core of the photosynthetic process is highly sensitive to environmental stresses. Its activity could be determined with one non-invasive time-resolved fluorescence measurement, which could measure the polyphasic rise with the basic steps of O-J-I-P (Havaux, 1992; Murata et al., 2007). Mathur et al. (2011) reported that PSII activity was reduced by 14% at 40°C relative to controls, and 45°C treatment induced irreversible damage to the oxygen-evolving complex in wheat (*Triticum aestivum*) leaves. Our previous research showed that high-temperature increased the trapped excitation flux per RC (TR_o_/RC) and the electron flux reducing end electron acceptors at the PSI acceptor side per RC (RE_o_/RC). In addition, the electron transport flux (further than Q^−^_*A*_) per RC (ET_o_/RC) was increased, which eventually caused inhibition of the overall PSII behavior (Chen et al., 2013). However, there is insufficient information about the existence of stress memory in the Photosystem II in response to stresses such as high-temperature.

Transcriptional memory of trainable genes was one important behavior character of stress memory (Ding et al., 2012). Recent studies have indicated that the metabolites were the end products of gene expression and the majority of the expressed genes could be activated by the metabolites at the post-transcriptional level (Guy et al., 2008; Shulaev et al., 2008). Therefore, metabolites abundance may reflect the regulation characteristic of stress memory. Previous studies have shown that the metabolites accumulation or distribution were involved in plant adaptation to environmental stresses (Rizhsky et al., 2004; Desbrosses et al., 2005; Du et al., 2011; Suzuki et al., 2012; Xu et al., 2013; Gai et al., 2014; Ruan, 2014). Gai et al. (2014) examined metabolic responses to phytoplasma infection in mulberry trees (*M. multicaulis* Perr.) using gas chromatography-mass spectrometry (GC-MS) analysis of polar compounds and found that 18 metabolites were detected exclusively in phytoplasma infection samples. These included three sugars, two sugar alcohols, two amino acids, four organic acids, two alcohols, three esters and lactones, and two nitrogen or sulfur compounds. Rizhsky et al. (2004) found that sucrose was elevated in *Arabidopsis* subjected to a combination of drought and high-temperature stress. In addition, Desbrosses et al. (2005) reported that legume nodules enriched 11 metabolites such as octadecanoic acid, asparagine, glutamate, homoserine, cysteine, putrescine, mannitol, threonic acid, gluconic acid, glyceric acid-3-P, and glycerol-3-P under plant-rhizobia interactions. Categorically, Du et al. (2011) and Xu et al. (2013) demonstrated that high-temperature enhanced significant changes in amino acid, carbohydrate metabolisms, organic acids. The special accumulation of those metabolites could trigger or inhibit regulation signal to increase the superior thermotolerance of cool-season turfgrass species.

High-temperature stress is one of major abiotic stresses limiting plant distribution, growth and productivity (Shah et al., 2011). Early studies demonstrated that the synthesis of heat shock proteins (HSPs) strengthened high-temperature response in plant (Howarth, 1991). HSPs could be grouped into three major families: low molecular weight HSP (LMW-HSP) ranging from 15 to 30 kDa, HSP70 ranging from 69 to 71 kDa, high molecular weight HSP (HMW-HSP) ranging from 80 to 114 kDa (Mian et al., 2008). Wang and Luthe (2003) reported that *LMW-HSP* gene such as *ApHsp26.2* and *ApHsp26.7a* highly accumulated in heat-tolerant creeping bentgrass variant than heat-sensitive ones. In addition, the superior thermotolerance in higher plants is correlated with *LMW-HSP* (*HSP18.1, HSP17.9*) expression in wheat (Basha et al., 1999) and *HMW-HSP* (*HSP101*) expression in *Arabidopsis* (Queitsch et al., 2000). However, whether *HSP* could be trainable or the working mechanism of the transcriptional memory in *HSP* is still undocumented.

Tall fescue (*Festuca arundinacea* Schreb.) is the predominant forage and cool-season turfgrass species grown widely in the temperate global regions. However, high temperature is a major factor limiting the growth of this turf species (Barnes, 1990; Zhang et al., 2005). In this study, heat tolerant PI 574522 and heat sensitive PI 512315, whose thermotolerance was identified through the summer adaptation test in Wu Han in 2010 and 2011, were used to investigate high temperature acclimation. Therefore, the objectives of this study were to: (i) investigate heat memory effects; (ii) determine the PSII photochemistry and metabolite responses induced by heat memory in tall fescue.

## Materials and methods

### Plant material and growth conditions

Single clonal plants of two tall fescue (*Festuca arundinacea* Schreb.) genotypes, heat tolerant PI 574522 and heat sensitive PI 512315 were transplanted from field plots to plastic pots field (13 cm diameter, 11 cm deep) filled with sand and peat soil (1/1, v/v) in the greenhouse under natural sunlight, temperatures of 22/18°C (day/night), relative humidity of 87% and wind speed of 0.8 m·s^−1^. Plants were maintained for 3 months to be established, and then transferred into controlled-environment growth chambers (HP300GS-C; Ruihua Instrument, Wuhan, China), with a 14-h photoperiod, photosynthetically active radiation at 450 μmol m^−2^ s^−1^ at the canopy level, a day/night temperature of 22/18°C and 70% humidity. Plants were allowed to acclimate for 1 week before heat treatments were imposed. Plants were watered 3 times weekly with half-strength Hoagland's solution (Hoagland and Arnon, 1950) until dripping during the entire experiment period in order to keep them close to field capacity.

### Treatments and experimental design

The plant-pot system was weighed at a 48-h interval to determine transpiration rate (T_r_) before starting heat treatment based on the method described by Hu et al. (2011). All plants for each tall fescue genotype were divided into three groups (Group I, II, III) and each group had four plant-pot systems with similar T_r_. Three treatment groups were assessed as the follows: (I) control, (II) pre-acclimation, and (III) non-acclimation. Heat acclimation (HA) was applied to plants in Group II (four pots). The plants in Group II were subjected to repeated trainable stresses as follows: plants were exposed to short HA treatments at 34°C for 4 h and annotated as stress 1 (S1), then placed in growth chambers with a temperature of 22°C for 20 h to get recovery and annotated as recovery 1 (R1). Stress 2 (S2) was achieved through subjecting R1 plants at 34°C for 4 h and moving S2 plants at 22°C for 20 h to get recovery 2 (R2). Stress 3 (S3), recovery 3 (R3), stress 4 (S4), and recovery 4 (R4) were achieved through above procedures shown in **Figure 2I**. To analyze gene expression, leaves were harvested at S0, R1, S2, R1, S3, R3, S4, and R4, respectively (**Figure 2I**). In addition, the plants in other two groups were grown in growth chambers under normal conditions as stated above. After 4 days, plants in Group II (pre-acclimation) and Group III (non-acclimation) of each genotype were exposed to prolonged heat stress (40/36°C at day/night) for 8 days. The leaf samples for physiology and gene expression analysis were collected at 0, 2, 4, and 8 d after treatment (DAT), respectively.

### Turf quality and cell membrane stability

Turf quality was assessed visually and recorded based on a 0–9 score system, in which 0 score indicates withered and yellow, thin and dead grass, 6 score is the minimum acceptable level according to color (percentage green leaves) of the grass, and 9 score indicates green, dense and uniform grass (Turgeon, 2002).

To determine electrolyte leakage (EL), about 0.1 g of fully developed leaves were excised to about 0.5 cm long segments, incubated in 15 mL deionized water, and shaken for 24 h at room temperature. The initial conductance (*C_i_*) of the incubation solution was determined using a conductance meter (JENCO-3173, Jenco Instruments, Inc., San Diego, CA, USA). Then, the test tubes were autoclaved at 120°C for 20 min to induce all electrolytes. The conductance of the incubation solution with killed tissues (*C*_max_) was determined after cooling down to room temperature. Relative EL (%) = (*C*_i_/*C*_max_) × 100.

### Chlorophyll a fluorescence transient measurements

Chlorophyll a fluorescence (OJIP) transient was measured by a pulse-amplitude modulation (PAM) fluorometer (PAM 2500, Heinz Walz GmbH) according to Li et al. (2010). All the measurements were done with 30 min dark-adapted leaves at room temperature. OJIP transients were induced by a red light pulse of 3000 μmol photons m^−2^ s^−1^ supplied by an array of light-emitting diodes. Excitation light of peak at 650 nm was focused on the surface of the leaves at a homogeneous spot about 5 mm in diameter to generate maximal fluorescence (Fm). The Chl a fluorescence emission measured and digitized at 10 μs intervals for the first 320 ms (Kautsky curve). There were 5–6 replicates per treatment.

### JIP test

OJIP transient was analyzed according to the JIP test. From OJIP transient, the extracted parameters (*F_o_, F_m_, F*_50μ*s*_, *F*_100μ*s*_, *F*_300μ*s*_, *F_J_, F_I_, M_o_* etc.) led to the calculation and derivation of a range of new parameters according to previous authors (Han et al., 2009; Chen et al., 2013) (see Table 1).

**Table 1:** **Parameters, formulae and their description using data extracted from chlorophyll (Chl) a fluorescence (OJIP) transient emitted by dark-adapted photosynthetic samples (Yusuf et al., 2010)**.

**Fluorescence parameters**	**Description**
**EXTRACTED PARAMETERS**	
*F_t_*	Fluorescence at time *t* after onset of actinic illumination
*F_o_*	Minimum fluorescence, when all PSII reaction centers (RCs) are open
*F_m_*	Maximum fluorescence, when all PSII RCs are closed
*F*_50μ_ 4_*s*_, *F*_100μ_ 4_*s*_, *F*_300_ 4_μ_ 4_*s*_	Fluorescence intensity at 50, 100, and 300 μs, respectively
*F_J_*	Fluorescence intensity at the J-step (2 ms)
*F_I_*	Fluorescence intensity at the I-step (30 ms)
Area	Total complementary area between fluorescence induction cure and *F* = *F*_m_
**TECHNICAL FLUORESCENCE PARAMETERS**	
V_t_4 = (F_t_ − F_o_)/(F_m_ − F_o_)	Relative variable fluorescence at time *t*
V_J_4 = (F_2ms_ − F_o_)/(F_m_ − F_o_)	Relative variable fluorescence at the J-step (2 ms)
V_I_4 = (F_30ms_ − F_o_)/(F_m_ − F_o_)	Relative variable fluorescence at the I-step (30 ms)
M_o_4 = 4 (F_300__μ_4_*s*_ – F_o_)/(F_m_ – F_o_)	Approximated initial slope (in ms^−1^) of the fluorescence transient V = f (t)
**QUANTUM YIELDS AND EFFICIENCIES/PROBABILITIES**	
φ_Po_4 = TR_O_/ABS = 1–(F_O_/F_m_)	Maximum quantum yield for primary photochemistry
ψ_Eo_4 = ET_O_/TR_O_4 = 1–V_J_	Effciency/probability with which a PSII trapped electron is transferred from Q_A_ to Q_B_
δ_Ro_ = 4RE_O_/ET_O_ = (1–V_I_)/(1–V_J_)	Efficiency/probability with which an electron from QB is transfered uuntil PSI acceptors
**SPECIFIC ENERGY FLUXES (PER ACTIVE PSII REACTION CENTER)**	
ABS/RC = M_o_(1/V_J_)(1/φ_Po_)	Absorbed photon flux per RC
TR_o_/RC = M_o_(1/V_J_)	Trapped excitation flux (leading to Q_A_ reduction) per RC
ET_o_/RC = M_o_(1/V_J_) ψ_Eo_	Electron transport flux (further than Q^−^_*A*_) per RC
RE_o_/RC = M_o_(1/V_J_) ψ_Eo_ 4δ4_Ro_	Electron flux reducing end electron acceptors at the PSI acceptor side, per RC

### RNA isolation, cDNA synthesis and real-time PCR assay

For RNA isolation, cDNA synthesis and real-time PCR assay, total RNA isolation and purification were performed according to the method described previously by Hu et al. (2013). For real time (RT)-PCR analyses, 2 μg purified RNA was converted into cDNA using cDNA synthesis kit according to the manufacturer's protocol (Fermentas, Burlington, ON, Canada). The expression level of the target genes was determined by real-time quantitative reverse transcriptase (RT)-PCR using SYBR Green Real-Time PCR Master Mix (Toyobo, Japan) and ABI StepOne Plus Real-Time PCR system (Applied Biosystems, Foster City, CA) in 20 μL reactions. Two-step RT-PCR procedure was performed in thermocycler conditions. In addition, the size of each amplified DNA fragment was verified on a 1.5% (w/v) agarose–ethidium bromide gel at 100 V for 40 min in 1 × TE buffer (10 mM Tris, 1 mM EDTA).

To determine the different responses at the transcriptional level in trained plants, four HSP genes [*HSC70, LMW-HSP* (about 18.8 kDa), *HMW-HSP* (about 98 kDa), *OsHSP74.8*] with different molecular weight were selected from 27 HSP genes based on the previous reports (Zhang et al., 2005; Mian et al., 2008; Zou et al., 2009; Giorno et al., 2010). The primer pairs used in this study were designed with Primer Premier software (Primer Premier v5.0; Premier Biosoft International, Palo Alto, Calif.) (Supporting Information Table S1). The relative expression of specific genes was quantified with comparative Ct method as described earlier (Li et al., 2005). *YT521-B* gene was used as a standard control in the RT-PCR reactions. The experiments were repeated twice with three replicates.

### Gas chromatography mass spectrometry (GC-MS) analysis

Metabolite profiles were determined with a GC–MS (DSQII, Agilent 7890A/5975C, Hemel Hempstead, USA) system based on the method described by Hancock et al. (2014), and samples metabolites were extracted based on the procedure reported by Roessner et al. (2000). A 1 μl of the derivatization solutions was injected into the DB5-MSTM column (15 m × 0.25 mm × 0.25 μm; J&W, Folsom, CA, USA). The inlet temperature was set at 280°C, the injection temperature was set to 290°C and the ion source temperature was adjusted to 200°C. The column temperature was initially kept a 5 min solvent delay at 70°C and then the GC oven temperature was raised to 260°C with 5°C min^−1^, and finally held at 260°C for 10 min. Helium was used as the carrier gas at a constant flow rate of 1 ml min^−1^. MS conditions: electron impact (EI) source, electron impact ionization (70 eV), solvent delay 4 min and the full scan mode (m/z 30–650) set at 0.6 scan s^−1^. The metabolites were identified using Agilent MSD Chemstation Software (version E.0200.493, Agilent Technologies, USA) coupled with NIST Mass Spectral Database (version 11). The experiments were repeated twice with four replicates. Each metabolite was identified based on the internal consistency of retention time (RT) and retention indices (RI). The retention indices were retrieved at the web sites http://gmd.mpimp-golm.mpg.de/search.aspx. Only metabolite detected at least three in five samples and held with internal consistency of RT and RI was considered true.

### Statistical analysis

The experiment was arranged in a completely randomized block design. Experiments were performed with four replicates (four plant-pot systems each treatment). Fisher's least significant difference test was applied for comparison between two means at *P* < 0.05 level. Analysis of variance was based on the general linear model procedure of SAS (version 9.0 for Windows; SAS Institute, Cary, NC).

## Results

### Heat stress-trained tall fescue plants

**Figure 1:**
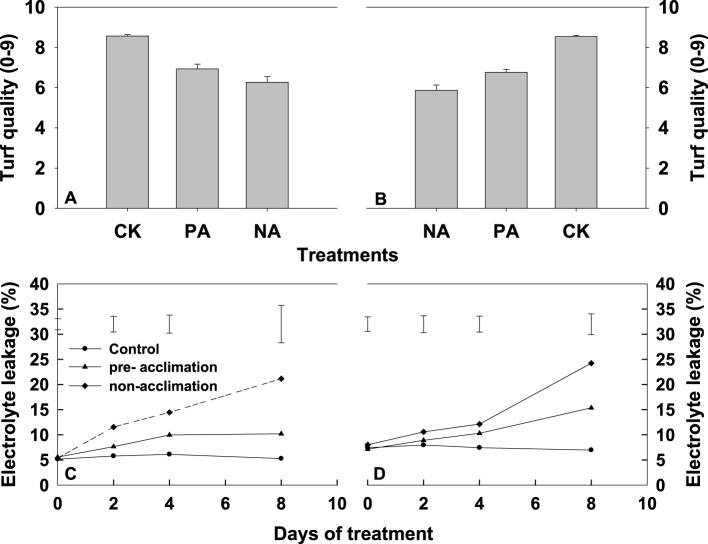
**Changes in turf quality (0–9) and electrolyte leakage (%) of PI 574522 (A,C) and PI 512315 (B,D) with increasing stressed time**. Plants were not stressed (CK), were heat stressed after high temperature pre-acclimation (PA) or without acclimation (NA). Turf quality score was determined at the end of this experiment. Vertical bars on the top indicate standard error **(A,C)**. Vertical bars on the bottom indicate protected-LSD values for treatment comparison at a given day of treatment **(B,D)**. Means were compared based on an LSD test at (*P* < 0.05).

To determine whether heat acclimation pretreatment decreased toxicity in tall fescue under heat stress, we measured turf quality and cell membrane thermostability (EL) in leaves of two tall fescue accessions under heat stress. During the entire 8-d treatment period, leaves of two tall fescue accessions exhibited severe decline in turf quality (Figures 1A,B) and increase in EL (Figures 1C,D). However, at the end of this experiment, both tall fescue genotypes in the pre-acclimation line had greater turf quality score than that in non-acclimation line (Figures 1A,B). Heat pre-exposed PI 574522 had the same level of EL as the control during whole experimental period, when subjected to heat stress (Figure 1C). The EL increased above the control in the heat pre-exposed PI 512315 subjected to heat stress at the end of experiment (Figure 1D). Under high temperature conditions, EL was greater for non-trained plants than for the control at the end of experiment, to 14.5% higher for PI 512315 than for PI 574522 (Figures 1C,D).

### Transcriptional memory from trainable and non-trainable HSP genes

**Figure 2:**
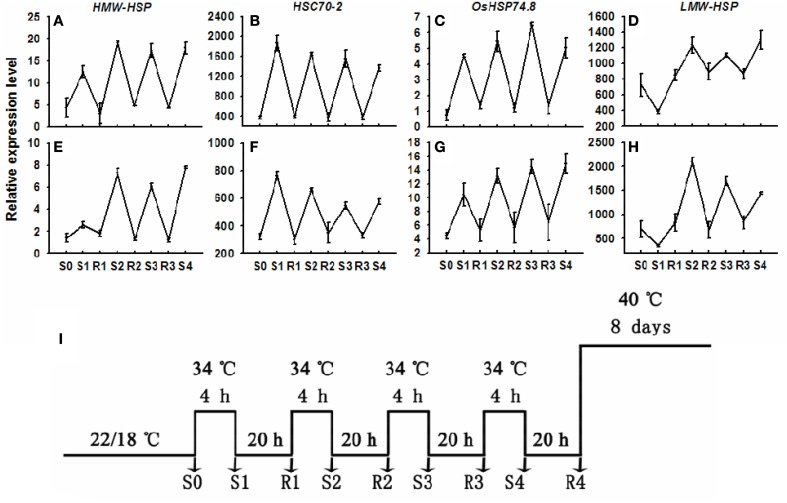
**Transcript levels of non-trainable and trainable genes in plants before and after single or multiple heat stresses**. RNA was isolated from plants in controls and from plants subjected to one to four heat stresses of 4 h of 34°C (S1–S4) separated by 20-h intervals of 22/18°C treatments (R1–R3). Transcript levels for **(A)** *HMW-HSP*; **(B)** *HSC70-2*; **(C)** *OsHSP74.8*; **(D)** *LMW-HSP* genes were measured by reverse transcription and real-time quantitative PCR. Genes are considered trained if the transcript level in S1 is considerably less than in subsequent stresses, as occurring for *HMW-HSP* and *LMW-HSP*. Experiments were repeated at least three times, each with three replicates, and the representative experiment shown indicates the mean ± se, *n* = 3 replicates.

Two distinct types of expression patterns were observed during repeated trainable stresses for both genotypes. The trainable genes, such as the *HMW-HSP* and *LMW-HSP*, showed considerably higher transcript levels during one or more subsequent stresses relative to the initial stress (Figures 2A,D,I; samples S1, S2, S3, and S4). In contrast, non-trainable genes, such as the *HSC70* and *OsHSP74.8*, repetitiously increased their transcripts to about the same level during each stress (Figures 2B,C,F,G; samples S1, S2, S3, and S4). Interestingly, both the trainable and the non-trainable genes in both genotypes returned to their initial (non-stressed) transcript levels during the recovery (22/18°C) states (samples R1, R2, and R3).

**Figure 3:**
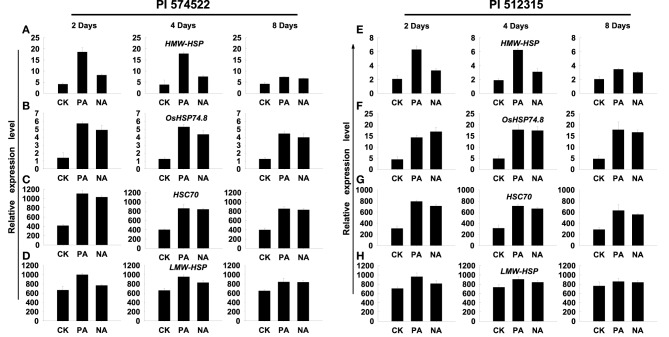
**Length of the transcriptional memory in trained plants**. The relative levels of mRNA of acclimated plants after recovery period under high temperature conditions for 2, 4, or 8 days, before and after a subsequent heat stress are shown in (**A–H**). Plants were not stressed (CK), were heat stressed after high temperature pre-acclimation (PA) or without acclimation (NA). Transcript levels of the trainable (*HMW-HSP* and *LMW-HSP*) genes. Transcript levels of the non-trainable (*HSC 70* and OsHSP 74.8) genes measured by reverse transcription–quantitative PCR (qPCR) in the leaves of PI 574522 **(A–D)** and PI 512315 **(E–H)** at 2, 4, and 8 days of heat stress. *YT-521B* was used as an internal control.

Next, we analyzed the period of transcriptional memory persistence in the absence of induced transcription. The trainable genes (*HMW-HSP* and *LMW-HSP*) showed increased transcript abundance in both pre-acclimation and non-acclimation lines of two tall fescue genotypes at 2, 4, and 8 DAT Figures 2A,D,E,H. However, trainable genes were induced higher transcript levels in all pre-acclimation lines than in all non-acclimation lines at 2 and 4 DAT, but not at 8 DAT (Figures 3A,D,E,H). Thereby, the duration of transcriptional memory could persist for up to 4 days in trained tall fescue plants. In addition, the non-trainable genes (*HSC70* and *OsHSP74.8*) showed increased transcript abundance in all treatment lines at 2, 4, and 8 DAT. However, no significant difference in the genes expression was observed between pre-acclimation and non-acclimation regime (Figures 3B,C,F,G).

### Characterization of leaf OJIP transients for pre/non-acclimation plants

**Figure 4:**
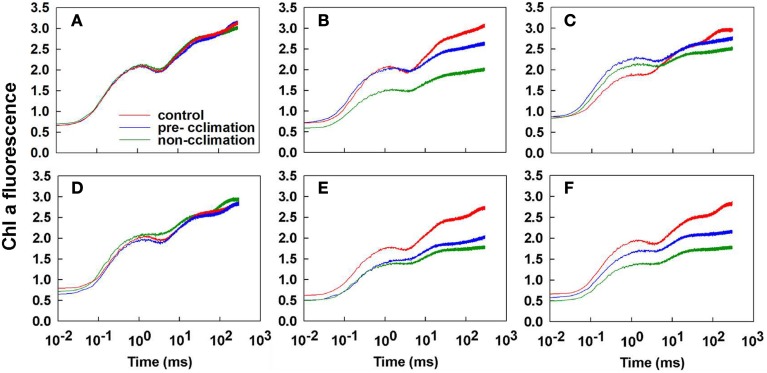
**Comparison of Chlorophyll a fluorescence (OJIP) transients of control lines (22/18°C day/night), and pre-accilimaton lines and non-accilimaton lines under heat stress (40/36°C at day/night) in tall fescue leaves**. OJIP transients were measured at 22°C and induced by 10 s light pulse of 3000 μmol photons m^−2^ s^−1^. Data for the JIP-test are sampled at 10 μs intervals for the first 320 ms. **(A–C)** present OJIP transients values in PI 574522 at 0, 2, and 8 HAT, respectively; **(D–F)** present OJIP transients values in PI 512315 at 0, 2, and 8 HAT, respectively.

In both tall fescue genotypes, the non-acclimated leaves exhibited different depression at the O-J-I-P step (Figure 4). Only the I- and the P-steps depression in the acclimated PI 574522 leaves was observed at 2 DAT (Figure 4). PI 574522 in the non-acclimation and pre-acclimation regimes had a large rise at the J- step and a large depression at the I- and the P-steps at 8 DAT. However, there was a greater depression at all steps of O-J-I-P for the acclimated or the not acclimated PI 512315 leaves at 2 and 8 DAT compared the control, to a greater decline the non-acclimation regime than in the pre-acclimation regime.

**Figure 5:**
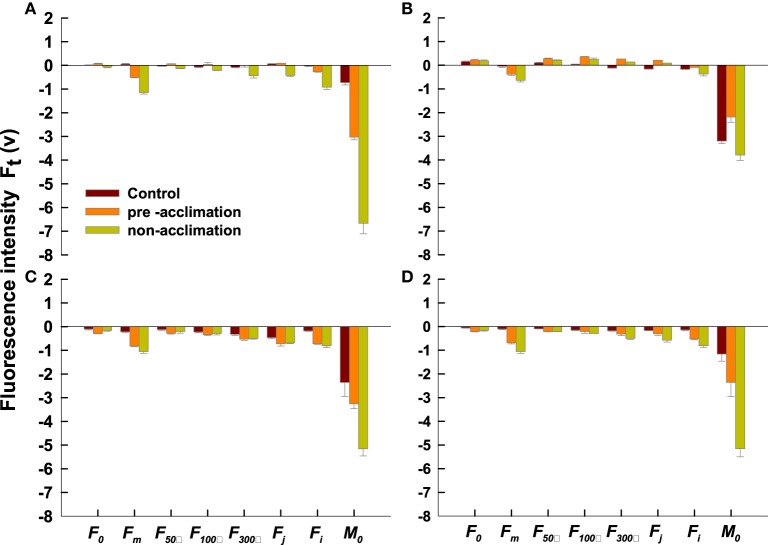
**Response to heat stress of pre-accilimaton or non-accilimaton plants reflected in data extracted from the recorded chlorophyll a fluorescence transient of tall fescue**. All the values were expressed relative to the sample treated at 0 d. Vertical bars represent means ± se (*n* = 5–6) based on least significant difference (LSD) test (*P* < 0.05). **(A,B)** present OJIP transients values in PI 574522 at 2 and 8 HAT, respectively; **(C,D)** present OJIP transients values in PI 512315 at 2 and 8 HAT, respectively.

Twelve fluorescence parameters were listed in Table 1. There was a lower level of F_o_, F_m_, F_50μs_, F_100μs_, F_300μs_, F_j_, F_i_, and M_o_ in the acclimation or the non-acclimation regimes for PI 574522 at 2 d compared to the control (Figure 5A). F_o_, F_50μs_, F_100μs_, F_300μs_, and F_j_ were greater for both acclimated or non-acclimated PI 574522 when compared to the control plants at 8 DAT (Figure 5B). However, when PI 512315 was subjected to high temperature for 2 DAT, F_o_ were much lower, but F_m_ and M_o_slightly decreased in the pre-acclimation vs. in the non-acclimation regime (Figure 5C). However, heat stress resulted in a decrease in F_o_, F_m_, F_50μs_, F_100μs_, F_300μs_, F_j_, F_i_, and M_o_ for acclimated and not acclimated PI 512315 at 8 DAT. F_m_, F_300μs_, F_j_, F_i_, and M_o_ were less decreased in the pre-acclimation regime vs. non-acclimation regime (Figure 5D).

**Figure 6:**
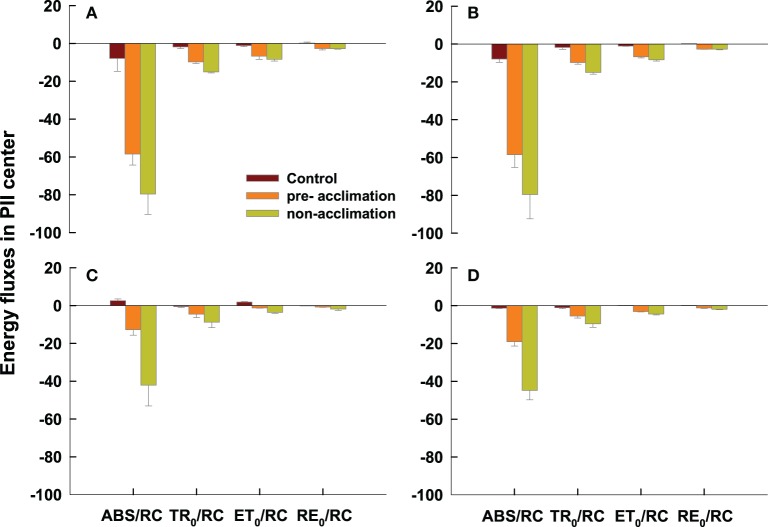
**Specific energy fluxes parameters in active PSII reaction center derived by the JIP-test from the average OJIP transients of Figure 2**. All the values were expressed relative to the sample treated at 0 d. Vertical bars represent means ± se (*n* = 5 – 6) based on least significant difference (LSD) test (*P* < 0.05). **(A,B)** present OJIP transients values in PI 574522 at 2 and 8 HAT, respectively; **(C,D)** present OJIP transients values in PI 512315 at 2 and 8 HAT, respectively.

Four energy fluxes parameters in active PSII reaction center declined in the pre-acclimation or non-acclimation regime, compared to the control. However, less decrease in ABS/RC and TR_0_/RC was observed in the pre-acclimation regime vs. non–acclimation for both genotypes at 2 and 8 DAT (Figure 6). Pre-acclimation line had a less decrease in ET_0_/RC than non-acclimation line at 2 and 8 DAT for PI 512315 (Figures 6C,D).

### Characterization of metabolic profiles for pre/non-acclimation plants

**Figure 7:**
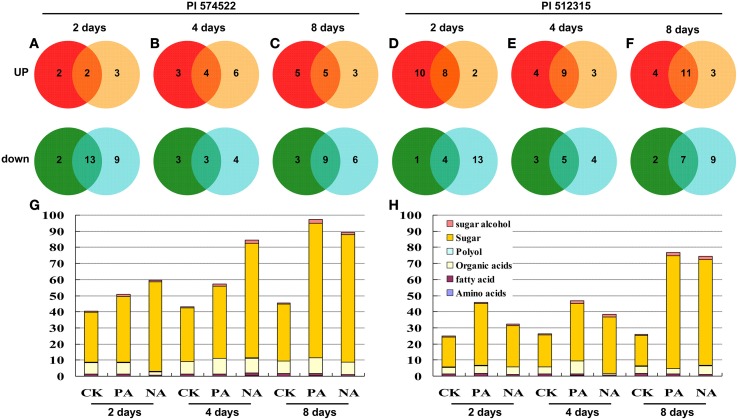
**Global view of the distinct and common metabolite targets in tall fescue plants in response to temperature shock**. Venn diagram of metabolites found in the GC-MS analyses between pre-acclimation line and non-acclimation line in the leaves of two tall fescue genotypes. The total number in each unique or overlapping set of metabolic analysis is shown, with graphical representation of functional categories for each set. **(A–C)** present fold change values relative to control in PI 574522 at 2, 4, and 8 DAT, respectively; **(D–F)** present fold change values relative to control in PI 512315 at 2, 4, and 8 DAT, respectively. **(G)** and **(H)** present metabolite distributions in PI 574522 and PI 512315, respectively.

The present metabolomic analysis provides a global view of commonly and differentially potential metabolites targets in trainable and non-trainable plants. Based on the internal consistency of retention time and retention indices, a total of 41 metabolites out of 160 total peaks with fairly consistent retention times and excellent resolution could be identified across all samples in this study (Table S2). The identified metabolites included 8 amino acids, 12 sugars, 15 organic acids, 4 fatty acids, and 2 polyols (Figure 7; Tables S3, S4). A Venn diagram analysis documented that different functional categories dominated the list of metabolites in tall fescue leaves subjected to the different treatment (Figure 7). Overall, 267 metabolites have significant concentration changes (*P* < 0.05) in response to high temperature between both tall fescue genotypes (Figure 7), of which 121 were detected in PI 574522 and 146 in PI 512315.

Heat-tolerant PI 574522 contained a number of up-regulated metabolites with the prolonged heat stress time in the acclimated plants, and the largest number (6) showed in non-acclimated plants at 4 DAT. Heat-sensitive PI 512315 had the largest number of unique up-regulated metabolites (10) in the acclimated plants at 2 DAT, and few up-regulated metabolites in non-acclimated plants exposed high-temperature. More unique up-regulated metabolites in PI 574522 were only detected in the acclimated than in non-acclimated plants at 8 DAT, while more unique up-regulated metabolites in trainable PI 574522 than in not acclimated plants for all regimes. Up-regulated metabolites of heat-tolerant PI 574522 consisted mainly of amino acids (3, 60%) at 8 DAT. However, for heat-sensitive PI 512315, up-regulated metabolites in the acclimated plants were mainly sugar (7, 41%). There were three up-regulated metabolites (threonic acid, proline, and citric acid) in both acclimated PI 574522 and PI 512315. Among the overlapping group of up-regulated metabolites identified in both trainable and non-acclimated plants, the number of metabolites was increased along with the increased stress time. And the number of overlapping up-regulated metabolites was less in PI 574522 than in PI 512315.

There were more unique down-regulated metabolites in not acclimated plants than in acclimated ones in both heat-stressed tall fescue genotypes (Figure 7). For heat-stressed PI 574522, down-regulated metabolites exclusively found in acclimated plants were mainly sugar (4, 50%). However, for heat-sensitive PI 512315, down-regulated metabolites exclusively found in acclimated plants were mainly organic acids (7, 54%). One sugar (lactulose) content decreased in both acclimated PI 574522 and PI 512315. For heat-tolerant PI 574522, there more common down-regulated metabolites in both acclimated and non-acclimated plants were detected at 2 (13 metabolites) and 8 (9 metabolites) DAT, relative to at 4 DAT (3 metabolites) (Figures 7A–C). In heat-sensitive PI 512315, the number of overlapping down-regulated metabolites was increased along with the increased stress time (Figures 7E–G). For heat-tolerant PI 574522, there were more metabolites unique in non-acclimated plants than in acclimated plants (12 vs. 10 for up-regulated; 19 vs. 8 for down-regulated).

## Discussion

In this study, we initially characterized the impact of memory from an earlier stress on *HSP* transcription, PSII photochemistry, and metabolites in high-temperature treated cool-season turfgrass. During repetitive exposures to high-temperature stress, the elevated transcriptional responses from *HSP* genes demonstrated the concept of a memory. Repetitive pre-exposures to multiple 4 h high-temperature stress and 20 h recovery treatments in a relatively short time (4 d) induced the stress memory in tall fescue, and enhanced the adaptability of high-temperature stress at the subsequent exposure. The present study disclosed that leaves turf quality score and EL in two tall fescue genotypes were lower in pre-acclimation regimes than that in non-acclimation regimes at 4 and 8 DAT. This observation confirmed previous findings that multiple dehydration stress/recovery pre-treatments significantly improved the physiological status of drought-stressed *Arabidopsis* leaf (Ding et al., 2012).

The high sensitivity of cool-season turfgrass to high-temperature stress is partly due to a defect in the accumulation of *HSP* transcription (Huang, 2003; Mian et al., 2008). As opposed to prolonged high-temperature treatment, our experimental system applied multiple high-temperature stress/recovery pre-acclimation which enabled observation and explanation of this high-temperature stress memory phenomenon in cool-season turfgrass. During the multiple high-temperature stress/recovery pre-acclimation stage, *HMW-HSP* and *LMW-HSP* obtained considerably higher transcript levels during one or more subsequent stresses (S2, S3, S4) relative to the initial stress (S1). They returned to their initial (non-stressed) transcript levels during the recovery (22/18°C) states (samples R1, R2, and R3), called trainable genes. The transcript abundance from the trainable genes displayed baseline transcript levels during the recovery states as well as more elevated transcript levels during the second treatments or on a following stress, which may be carried forward as the concept of “transcriptional memory”. The transcriptional memory, as one effects of stress memory that was activated by the previous day's high-temperature stress may be essential in adapting subsequent stress conditions in plants (Francis and Kingston, 2001; Ohi et al., 2011).

In addition, we found that the trainable genes (*HMW-HSP* and *LMW-HSP*) were only induced at higher transcript levels in all pre-acclimation lines than in all non-acclimation lines at 2 and 4 DAT in two tall fescue genotypes in post-trainable treatment phases. This indicates that the transcriptional memory could persist for up to 4 days in pre-acclimation tall fescue plants. These data also point out that the transcriptional memory in the trainable genes (*HMW-HSP* and *LMW-HSP*) can continue to act as positive regulation signals in plants' response to subsequent high-temperature stress. Moreover, the trainable genes activated different regulation signals in tall fescue genotypes with different thermotolerance capacities. *HMW-HSP* and *LMW-HSP* showed similar transcript abundance between trainable and post-trainable phases in heat-stressed PI 574522. However, in heat-stressed PI 512315, the expression level of *HMW-HSP* and *LMW-HSP* was less in post-trainable phase than that in trainable phase. This declined expression level during the post-trainable phase was similar to *HsfA2* and *Hsp17-CII* expressing in trainable tomato (*Lycopersicon esculentum*) anthers in response to high-temperature (Giorno et al., 2010). The current data further disclosed that activated transcriptional memory had spatio-temporal effects to facilitate the plant's response to high-temperature stress.

Our previous research has shown that high-temperature stress caused less electron transport or less energy exchange between independent photosystem II (PSII) units in tall fescue leaves (Chen et al., 2013). Our question was how the activated stress memory regulated the recovery process of PSII. To address this question we analyzed the OJIP fluorescence transient in pre/non-acclimation grass leaves by JIP-test in this study. As predicted, except at 2 DAT in PI 512315, all pre-acclimation leaves showed less severe decline at steps of O-J-I-P than that in non-acclimation leaves at 2 and 8 DAT for both tall fescue genotypes. Subsequently, *F_m_* and *M_o_*were less decreased by pre-acclimation vs. by non-acclimation, indicating that the stress memory effects can alleviate high-temperature toxicity on PSII in cool-season turfgrass. In per active PSII RCs, absorbed photon flux per RC (ABS/RC), trapped excitation flux (TRo/RC), electron transport flux (further than Q^−^_A_) (ETo/RC) as well as the electron flux reducing end electron acceptors (RE_o_/RC) acted synergistically to reduce the electron acceptor Q_A_ to Q^−^_A_. The converting of excitation energy to redox energy, ultimately led to CO_2_ fixation (Strasser et al., 2000). The specific energy fluxes in per active PSII RC were sensitive to high-temperature stress (De Ronde et al., 2004; Wen et al., 2005). In this study, we also found that ABS/RC and TR_0_/RC were less decreased by pre-acclimation than by non-acclimation for both genotypes at 2 and 8 DAT. In addition, plants in pre-acclimation line had a less decrease of ET_0_/RC than those in non-acclimation line at 2 and 8 DAT for PI 512315. These results further validated that stress memory could contribute to slow down the high-temperature induced CO_2_ fixation damage from inhibited energy transport fluxes in active PSII RC.

Plant metabolites, such as soluble sugars, amino acids, organic acids, and lipids, are sensitive to high-temperature and are critical to high-temperature stress adaptation (Du et al., 2011; Hancock et al., 2014). This study avails pioneer information about the role of metabolites profiles in stress memory in response to high-temperature stress in cool-season turfgrass. Metabolites were exclusively up-regulated in trainable plants, involving four organic acids, eight sugars, five amino acids, and one fatty acid. The tricarboxylic acid cycle is critical in energy metabolism through generating reducing agents and ATP (Rolo and Palmeira, 2006; Nunes-Nesi et al., 2013). The higher levels of TCA intermediates under environmental stress conditions, such as citric, malic, and isocitric acids, could be reflective of greater mitochondrial activity, which is directed toward generating more energy (Vasquez-Robinet et al., 2008; Du et al., 2011). Tris phosphoric acid could supply adequate P secondary compounds for cell energy metabolism. Threonic acid is a major product from ascorbic acid metabolism, which could induce the essential protective effects in plant defense against oxidative stress (Navascués et al., 2012). Kaplan et al. (2004) reported that all the contents of citric acid, malic acid and threonic acid increased in *Arabidopsis* exposed to heat stress. However, citric acid, malic acid and threonic acid showed significant decrease at 2 days of heat stress for PI 512315 with no previous high-temperature acclimation, while was up-regulated in pre-acclimation grass at 2 DAT. The content of tris phosphoric acid decreased in the non-acclimation grass at 2 DAT. Furthermore, citric acid was up-regulated in pre-acclimation PI 574522 at 2 DAT, but down-regulated in non-acclimation grass at 2 and 4 DAT. In 2 and 4 days stressed PI 574522, pre-acclimation leaves contained higher total content of threonic acid than did non-acclimation ones. The accumulation of the three organic acids in tall fescue in post-trainable treatment phases might reflect a stress memory response to high-temperature stress to produce more energy or ascorbates for copying with heat-induced oxidative stress.

A signature feature of the trained plants was the changes of carbohydrates metabolites during their prolonged high-temperature stress periods. Soluble sugar is the major form of carbohydrates metabolites and signal molecule, and key for plant organism structure and cell metabolism (Farrar et al., 2000; Hu et al., 2013). Our finding showed that the significantly higher content of idose and allose was detected in trained plants but unchanged in non-trained ones for heat-tolerant PI 574522 at 4 DAT. Similarly in heat-sensitive PI 512315, allose and talose content increased in trained plants but unchanged in non-trained ones at 4 DAT. The up-regulated rare monosaccharides (idose, allose, and talose) in pre-acclimation plants could contribute to its superior heat tolerance, as rare monosaccharides accumulation acts as a triggering molecule of plant defense (Kano et al., 2013). When the grass was exposed high-temperature for 8 days, the elevated levels of three rare monosaccharides in pre-acclimation plants were also detected. This was inconsistent with the loss of the transcriptional memory at the trainable genes by day 8. Therefore, despite the absence of transcriptional memory of the trainable genes, a trained stress memory response of carbohydrates metabolites is still displayed. Sucrose is the major form of carbohydrates in the resource allocation system in most plants (Zimmermann, 1957). Hexoses (i.e., glucoheptose and glucose) produced from the sucrose digest are important signal molecules in source–sink regulation, which can act as regulators of gene expression (Savitch et al., 2002; Hu et al., 2013). The increased sucrose and hexoses content in pre-acclimation heat-sensitive genotype may be another potential mechanism for stress memory in response to heat stress. In pre-acclimation heat-tolerant genotype, sucrose may be vital in stress memory defense mechanism.

Evidence has shown that the amino acid could act as signaling molecule to regulate transcription from a large number of transcription factors and has a critical role in the stress responses (Harding et al., 2000, 2003; Sharma and Dietz, 2006). In this study, proline, serine, and pyroglutamic acid were exclusively up-regulated in pre-acclimation PI 574522 at 8 DAT. Proline and glycine were exclusively up-regulated in pre-acclimation PI 512315 at 2 DAT. The accumulation of proline was known to be associated with stress conditions involving osmotic stress and high-temperature stress (Ashraf and Foolad, 2007; Cvikrová et al., 2012). In this study, we demonstrated that the pre-acclimated plants exhibited higher cell membrane stability (lower EL level), and the lower EL level may be attributable to the greater accumulation of proline in pre-acclimation plants. In the pre-acclimation lines, the earlier increases in proline for heat-sensitive PI 512315 than that in heat-tolerant PI 574522 during the prolonged high-temperature stress stage indicated that the stress memory effects of proline metabolism was relative with the sensitivity of this species to high-temperature stress. Subsequently, we inferred that the exclusive increased content of serine, pyroglutamic acid and glycine in pre-acclimation PI 574522 and PI 512315 respectively reflected the sensitivity of tall fescue species to high-temperature stress. Seven amino acids (valine, serine, proline, glycine, alanine, pyroglutamic acid, glutamic acid) increased at 4 DAT, and two amino acids (pyroglutamic acid, proline) increased at 8 DAT for both trained and non-trained PI 512315. However, for both trained and non-trained PI 574522, six amino acid (alanine, serine, glycine, alanine, pyroglutamic acid, glutamic acid) were down-regulated at 2 DAT, two kinds (alanine, glutamic acid) decreased at 4 DAT, and one kind (glutamic acid) decreased at 8 DAT. The increased amino acids in heat-stressed PI 512315 but decreased in heat-stressed PI 574522 demonstrated further that the different metabolism response of amino acid was relative with the sensitivity of this species to high-temperature stress.

Furthermore, five kinds of fatty acid (butanoic acid, hexadecanoic acid, octadecan, octadecadienoic acid, octadecanoic acid) changed in the pre/non-acclimation tall fescue, which may be also partly responsible for stress memory response to high-temperature stress. For example, the higher thermotolerance in pre-acclimation PI 512315 may be due to the increased content of butanoic acid, and the lower thermotolerance in non-acclimation PI 512315 may be due to the decreased content of hexadecanoic acid, octadecanoic acid, and octadecan. However, the significant changes of these fatty acids were not detected in pre-acclimation PI 574522. Interestingly, the octadecadienoic acid content increased in non-acclimation PI 574522. C_16_, C_18_, and C_20_ fatty acids are key in plant cell membrane in response to biotic and abiotic stresses (Di Pasqua et al., 2006; Grausem et al., 2014). C_16_ and C_18_ fatty acids such as hexadecanoic acid, octadecanoic acid, and octadecan acid changed in non-acclimation PI 512315 and PI 574522, which may reflect their cell membrane injure under high-temperature stress.

## Conclusion

In conclusion, our results showed that pre-acclimation treatment could induce stress memory involved in *HSP* transcriptional memory, PSII photochemistry and metabolic responses. This provides novel insights into the high-temperature acclimation process for cool-season turfgrass. The transcriptional memory from two trainable genes (*HMW-HSP* and *LMW-HSP*) was activated in the multiple acclimation stage and could be persistent under prolonged high-temperature conditions for 4 days. The heat pre-acclimation treatment could improve the recovery process of PSII by declining down the inhibition of energy transport fluxes in active PSII RC. The stress memory obtained from heat acclimation pretreatment has a greater effect on the metabolite profiles in pre-acclimation tall fescue, and different metabolic response including organic acids, sugars, amino acids, and fatty acid. These findings will be particularly useful in comprehending the key contribution of stress memory in heat response mechanisms, and improving the novel response strategies for a cool-season turfgrass's ability to respond to the high-temperature environment.

## Author contributions

JF and TH conceived and designed the experiments. TH and SL performed the experiments. TH analyzed the data. TH and EA wrote the manuscript. All authors read and approved the final manuscript.

### Conflict of interest statement

The authors declare that the research was conducted in the absence of any commercial or financial relationships that could be construed as a potential conflict of interest.
